# Why is change a challenge in acute mental health wards? A cross‐sectional investigation of the relationships between burnout, occupational status and nurses’ perceptions of barriers to change

**DOI:** 10.1111/inm.12517

**Published:** 2018-07-11

**Authors:** Caroline Laker, Matteo Cella, Felicity Callard, Til Wykes

**Affiliations:** ^1^ Department of Psychology Institute of Psychiatry, Psychology & Neuroscience King's College, London London UK; ^2^ Anglia Ruskin University Chelmsford UK; ^3^ Birkbeck, University of London London UK; ^4^ South London & Maudsley NHS Foundation Trust Bethlem Royal Hospital Beckenham UK

**Keywords:** mental health wards, nursing, opinions, organizational change

## Abstract

Changes in UK psychiatric wards have been difficult to implement. Specific areas of nursing staff resistance remain unclear. Previous healthcare research suggests that burnout is common and that managers’ regard changes more positively than direct care staff. We will therefore examine whether burnout and workforce characteristics influence psychiatric nurses’ perceptions of barriers to change. Psychiatric nurses (*N* = 125) completed perceptions measures of ‘barriers to change’ (VOCALISE: subscales included ‘powerlessness, confidence and demotivation’); and ‘burnout’ (Maslach Burnout Inventory: subscales included ‘emotional exhaustion, personal accomplishment and depersonalization ‘). Staff characteristics, such as length of employment, occupational status, education, ethnicity, gender and age, were also collected. Correlations between these measures informed random‐effects regression models, which were conducted to predict the barriers to change score and to explore differential effects in the subscales of VOCALISE. Perceptions of barriers to change (VOCALISE) were correlated with burnout (*r* = 0.39)*,* occupational status (*r* = −0.18) and age (*r* = 0.22). Burnout (Coef. β: 10.52; *P* > 0.001) and occupational status (Coef. β: −4.58; *P* = 0.05) predicted VOCALISE. Emotional exhaustion (Coef. β: 0.18; *P* < 0.001) and low personal accomplishment (Coef. β: 0.21; *P* = 0.001) predicted powerlessness. Emotional exhaustion predicted low motivation regarding changes (Coef. β: 0.11; *P* = 0.005). Low confidence predicted high levels of depersonalization (Coef β: 0.23; *P* = 0.01). Direct care staff expressed significantly more powerlessness (Coef. β: −2.60; *P* = 0.02) and significantly less confidence (Coef. β: −3.07; *P* = 0.002) than managers. For changes to be successful in psychiatric wards, burnout will need to be addressed. Future change strategies may consider involving direct care staff to improve perceptions of barriers to change.

## Introduction

Although adapting to organizational change is a necessary aspect of working life, many employees find changes stressful (Lewin 1951; Schein 1996). In UK NHS hospitals, and internationally, staff face numerous organizational uncertainties as a result of resource shortages (e.g. increasing reliance on agency staff) that might influence how they view upcoming changes (Aiken *et al*. 2002; Cleary *et al*. 2010; Thompson *et al*. 2008; Yadav & Fealy 2012). In mental health hospitals, there are additional factors such as acute mental distress and service user aggression, which might also contribute to how readily staff accept changes (Kindy *et al*. 2005; McGeorge *et al*. 2001). Changes have been difficult to embed in mental health wards, with service users reporting limited improvements (Evans *et al*. 2012; MHAC 2005), and insufficient therapeutic activity of a psychological nature (e.g. psychosocial interventions, cognitive behavioural therapy, solution‐focused behavioural therapy and family therapy; Csipke *et al*. 2014; Sharac *et al*. 2010). It is therefore clear that negative influences on staff perceptions of barriers to change need to be identified. This will allow focused change management strategies to be developed.

In mental health settings, burnout is an international problem (Morse *et al*. 2012). Globally, burnout has negative impacts on the workplace experience for nurses, affecting staff retention and the clinician/client relationship (Hanrahan *et al*. 2010; Morse *et al*. 2012). In the United Kingdom, burnout is higher in mental health settings than in other hospital settings (Johnson *et al*. 2011; Totman *et al*. 2011). It is therefore important to investigate whether burnout influences how staff respond to changes. Although there is no evidence exploring the relationship between burnout and staff perceptions towards changes in adult mental health, previous research shows that attitudes towards implementing changes in practice have worsened in the presence of burnout in Swedish neonatal units and in US children's mental health services (Aarons & Sawitzky 2006; Wallin *et al*. 2006). Examining whether burnout contributes to negative perceptions of barriers to change in UK mental health wards may help to explain why some staff resist changes.

There may also be workforce factors that influence how change is appraised and implemented. The wider, organizational management literature suggests that younger corporate employees may regard changes positively, being more likely to take risks and try new strategies (Vroom & Pahl 1971). Racial and gender diversity has shown mixed effects either by enhancing innovative behaviour (Van der Vegt & Janssen 2003) or by limiting performance (Baugh & Graen 1997). In children's mental health care, in the United States, staff with higher educational attainment have shown more positive responses to evidence‐based practice (EBP) changes; and those with greater clinical experience (i.e. a longer length of employment) have responded more negatively to new approaches, compared to interns who were more open towards change (Aarons 2004). In the United Kingdom, NHS managers who participated in a safer patient initiative (in general healthcare work areas, including medicines management, critical care, perioperative care and general ward care) and managers from mental health acute in‐patient wards have shown more positive views of changes than staff on the front line (Benn *et al*. 2009; Laker *et al*. 2012). If there are workforce characteristics which affect how staff respond to change, this information might usefully inform future change strategies (i.e. how to allocate support and resources).

## Aims

This study uses cross‐sectional data from nursing staff working within seven inner city, acute in‐patient wards, and a specialist in‐patient women's service, in a mental health NHS Foundation trust.

We will explore the effects of staff burnout and workforce characteristics on perceptions of barriers to change. This is important because identifying specific areas of resistance will allow targeted change strategies in the future.

We will focus on the perceptions of nurses (qualified and support staff) because nurses are the largest group of employees in the NHS (Horan *et al*. 2015), and they have the potential to play a vital role in promoting changes.

There are three aims: (i) to examine whether burnout predicts perceptions of barriers to change; (ii) to explore the relative contribution of two potential influencers of barriers to change (burnout and workforce characteristics). If these variables show independent effects, the subscales will be analysed to explore; (iii) how perceptions of barriers to change (VOCALISE subscales: powerlessness, confidence, demotivation) differ amongst significant staff groups, and how the subscales of the Maslach Burnout Inventory (emotional exhaustion, depersonalization, personal accomplishment) are related to the subscales of the VOCALISE measure. These details may help to identify future areas for support/training.

## Methods

### Study design

Data were collected as part of a randomized controlled trial (entitled: DOORWAYS), which aimed to improve the therapeutic milieu in eight acute in‐patient wards by introducing predominantly nurse‐led therapeutic interventions (Trial Registration: ISRCTN 06545047; Wykes *et al*. 2018). A local NHS Research Ethics Committee (07/H0809/49) awarded ethical approval for this study.

### Sample size

As there are no studies which examine relationships between perceptions of barriers to change and ward variables, the sample size was based on the need to detect a relevant correlation with a specified significance level and power (Lachin 1981). The sample size required to detect a correlation of 0.3 with 90% power using a two‐sided test of the null hypothesis that the correlation is 0 and assuming a 5% significance level was *N* = 113. To estimate the number of participants necessary for multilevel regression models, we followed the general rule suggested by Green (1991) of ten cases per variable.

### Participants

All permanently employed ward nursing staff were eligible to take part (*N* = 154), including staff from band seven (team leaders), band six (clinical charge nurses), band five (entry level qualified staff) and band three (healthcare assistants). Temporary staff and student nurses were excluded.

### Procedure

Staff (*N* = 125) were recruited over 4 weeks. An on‐site team of research assistants asked ward staff to consider participation, and participants provided written, informed consent. Staff completed measures of perceptions of barriers to change (VOCALISE) and burnout (Maslach Burnout Inventory) both of which included three subscales, and they provided demographic information.

### Measures

#### Staff perceptions of barriers to change

We developed the Views Of Change and Limitations in In‐patient Settings (VOCALISE) measure (Laker *et al*. 2014) using a method of stakeholder participation to allow ‘real setting’ items to emerge. The items describe emotional/psychological responses to change. The psychometric properties of VOCALISE are described in another paper (Laker *et al*. 2014). A factor analysis of the 18‐item measure allowed three subscales to be developed, which include powerlessness (seven items), confidence (six Items) and demotivation (five items). These subscales will allow an exploration of socio‐psychological factors, which may shape how staff view changes. The VOCALISE measure can be accessed at www.perceive.iop.kcl.ac.uk. In this study, scores higher than the median (63) will indicate negative perceptions.

#### Staff demographics

The staff variables were handled as two‐level factor variables to allow post hoc comparison of marginal means (e.g. gender: male/female). They included the following:

- Length of employment (less than 41 months/more than 42 months).
- Occupational status (direct care staff/senior staff).
- Education (no degree/degree educated).
- Ethnicity (BME/White British).
- Gender (female/male).
- Age (less than 39 years/more than 40 years).

Variables based on continuous data (e.g. burnout, length of employment and age) were split at the median to create two levels (lower/higher). The occupational status variable captured staff grade, dichotomized into two groups. Managerial staff included team leaders and band six nurses. The direct care staff group included healthcare assistants and band five nurses.

#### Burnout

Maslach Burnout Inventory (Maslach *et al*. 1996). This measure captures personal accomplishment, emotional exhaustion and depersonalization (cynicism). It has been used widely in the field of mental health nursing. Although there are alternative measures of stress such as the Perceived Stress Scale (Cohen *et al*. 1983), and the COPE Inventory (Carver 1997), these measures are global. The MBI was preferred as the items are focused on the views of clinicians in workplace settings, and take the relationship between clinician and client into consideration which is crucial in mental health nursing.

In this study, the continuous MBI measure was split at the median to create two categories (low scores/high scores) for ease of comparison with VOCALISE, and because there were limited numbers of participants on each ward.

### Analysis strategy

Associations between perceptions of barriers to change, burnout and workforce characteristics were examined using Pearson's and partial correlations (Mayers 2013). Pearson's correlations were used to identify which variable was most closely associated with VOCALISE. Partial correlations were used to ensure that variables with a unique contribution to VOCALISE were included in the regression model.

The regression modelling took into consideration the relative impact of the predictor variables on VOCALISE (staff perceptions of barriers to change). This was important because there are resource constraints in the UK national health services. Identifying high impact issues will ensure viable change management strategies. A two‐stage random‐effects regression model was therefore developed, omitting variables that were weakly correlated with VOCALISE (*r* = 0.1 or smaller), as these are considered insubstantial (Cohen 1988). In stage one, the explanatory effect of the variable most strongly correlated with VOCALISE, which was burnout (MBI), was explored. In stage two, any additional effects of uniquely correlated workforce characteristics were examined.

Hypothesis 1: The contribution of burnout to perceptions of barriers to change will be greater than the contribution of workforce characteristics.

Random‐effects models were suitable because they include an added variance component to explain an additional level of variation in the hierarchical (multilevel) data structure. The data used in this study required such an approach because the perceptions of individuals were likely to be related because they come into contact on the ward, which was included as a cluster variable. In these models, σ_u_ corresponds to the standard deviation of the average total score of the dependent variable across wards; σ_e_ is a measure of the ‘unobserved variance’ or the residual standard deviation within wards and rho measures the percentage of variability in the dependent variable's total scores due to ward heterogeneity (Vittinghoff *et al*. 2005). These statistical parameters are indicators of variation in the data.

Post hoc analyses were conducted (using Stata command *margins),* after each random‐effects model to show how the groups in the independent variables were related to the dependent variable. This was achieved by predicting a mean VOCALISE score based on each of the groups outlined in the measures section (e.g. for occupational status, the groups are ‘direct care staff/senior staff’. All analyses were conducted using STATA 14, StataCorp LLC, Texas, USA.

Variables that had a significant effect on VOCALISE were retained to explore whether differential effects existed in the VOCALISE subscales (powerlessness, confidence and demotivation).

## Results

### Sample

Staff of all grades (*N* = 125) participated across eight acute in‐patient wards (Table 1). On each ward, the completion rate was between 65 and 100% of all available nursing staff per ward. Table 1 highlights service characteristics. Seven wards were acute mental health wards. Ward 4 was a specialist service run by women for women experiencing a mental health crisis; hence, there were no male nurses present. Generally, there were fewer men represented in the sample because there are fewer male nurses employed on the wards than female nurses. Across the wards, the ethnicity and age of staff were similar.

**Table 1:** Workforce characteristics

Wards		1	2	3	4	5	6	7	8	Total (%)
*N*	No. of staff	18 (15)	13 (10)	16 (12)	8 (6)	19 (15)	15 (12)	18 (15)	18 (15)	125 (100)
Staff Grade	HCA	7	3	6	1	5	4	7	6	39 (31)
	Band 5	8	7	7	3	12	8	7	6	58 (47)
	Band 6	1	2	1	3	1	0	3	4	15 (12)
	Band 7	1	1	1	1	1	1	1	1	8 (6)
	Missing	1	0	1	0	0	2	0	1	5 (4)
Ethnic Group	White British/Other	6	2	3	4	5	3	4	6	33 (27)
	BME	12	11	12	4	14	12	14	10	89 (71)
	Missing	0	0	1	0	0	0	0	2	3 (2)
Gender	Male	3	7	12	0	9	3	9	3	46 (37)
	Female	15	6	4	8	10	12	9	15	79 (63)
Age	Mean (SD)	39.63 (13.0)	36.38 (7.61)	38 (7.93)	44.25 (4.80)	43.26 (9.94)	35.38 (8.82)	39.6 (8.61)	40.07 (9.85)	39.57
	Max/min	27–50	22–62	24–55	37–49	26–67	22–48	27–55	23–54	N/A

### Correlations

Pearson's correlations were computed to explore any associations between perceptions of barriers to change (VOCALISE) and the potential predictors: burnout, length of employment, occupational status, education, ethnicity, gender and age. Table 2 shows a moderate, positive correlation between perceptions of barriers to change and burnout. Occupational status and age were weakly correlated. Those in direct care positions and younger staff had more negative perceptions of barriers to change than managers and older staff.

**Table 2:** Correlations: workforce characteristics and burnout with VOCALISE

	MBI	Length of employment	Occupational status	Education	Ethnicity	Gender	Age
VOCALISE	***r = 0.39***	***r*** ** ** ***=*** ** **0.001	***r = −0.18***	***r*** ** ** ***=*** ** **0.03	***r*** ** ** ***=*** ** **0.17	***r = −***0.001	***r = −0.22***
	***P*** **=** ***>0.001***	***P*** ** = **0.99	***P = 0.05***	***P*** ** = **0.74	***P*** ** = **0.06	***P*** ** = **0.99	***P = 0.02***
	***n = 114***	***n*** ** = **111	***n = 119***	***n*** ** = **97	***n*** ** = **119	***n*** ** = **122	***n = 110***

Bold and italics values denote significant findings.

Table 3 shows that after taking the effects of burnout into consideration, the association between perceptions of barriers to change and occupational status remained. Age became less closely correlated, indicating that much of the correlation between age and VOCALISE was due to the correlation between age and burnout.

**Table 3:** Partial correlations of VOCALISE with burnout, occupational status and age

Variables (*n* = 101)	Partial Corr. (*r*)	*P* value
Burnout (MBI)	0.36	>0.001
Occupational status (manager/direct care staff)	*−*0.25	0.01
Age (39 years/40+)	*−*0.08	0.40

Managerial staff included team leaders and band six nurses. The direct care staff group included healthcare assistants and band five nurses.

### Random‐effects models

#### Model 1

##### A two‐stage model showing the contribution of burnout, occupational status and age in explaining perceptions of barriers to change

Given burnout was the variable most strongly correlated with perceptions of barriers to change, it was included in the first stage. Age and occupational status were added in the second stage to explore whether these workforce characteristics and burnout explained shared variance in perceptions of barriers to change (VOCALISE; Table 4).

**Table 4:** What is the effect of burnout on perceptions of barriers to change (Stage 1)?

Variables	Coefficient beta (Coef. β)	Standard error (SE)	*P* value (Statistical significance)	95% Confidence interval (C.I.)	
				Upper limits (UL)	Lower limits (LL)
Burnout	10.58	1.83	>0.001	7.00	14.17
_cons	57.39	1.30	0.00	54.84	59.95

*N* = 114; σ_u_ = 0; σ_e_ = 9.43; ρ = 0.

In this model (χ^2^(1) = 33.5; *P* > 0.001), burnout explained 23% of the variance in the VOCALISE measure (perceptions of barriers to change). Staff with higher burnout had more pessimistic perceptions of barriers to change (predicted mean VOCALISE score: 67.98). Staff with lower burnout scores had more optimistic views of change (predicted mean VOCALISE score: 57.39; Table 5).

**Table 5:** What is the shared effect of burnout, occupational status and age on perceptions of barriers to change (Stage 2)?

Variables	Coef. (β)	SE	*P* Value	95% CI	
				Upper limits (UL)	Lower limits (LL)
Burnout	10.52	1.92	>0.001	6.76	14.27
Age	−2.81	1.91	0.14	−6.54	0.93
Occupational status: manager/direct care staff	−4.58	2.31	0.05	−9.11	−0.06
_cons	59.41	1.76	0.00	55.96	62.86

*N* = 101; σ_u_ = 0; σ_e_ = 9.17; ρ = 0.

This model (χ^2^(3) = 43.20; *P* > 0.001) shows that burnout and occupational status significantly affected perceptions of barriers to change, accounting for 30.8% of the variance in the VOCALISE variable. As the effects of occupational status and burnout on perceptions of barriers to change appeared to be independent (with no meaningful change in the beta scores), there was no change to the predicted mean burnout scores. Staff in direct care positions had more negative perceptions of barriers to change (predicted mean VOCALISE score: 63.31). Those in managers’ positions were more positive towards changes (predicted mean VOCALISE score: 58.72).

##### Subscales (models 2, 3 & 4)

The effects of significant variables on perceptions of barriers to change (VOCALISE) were examined further to explore how those in direct care groups expressed barriers to change, according to the subscales of VOCALISE. Any differential effects of the subscales of the Maslach Burnout Inventory on perceptions of barriers to change were also considered. Three models were tested as follows:

- Model 2: Dependent variable (dv): VOCALISE: Powerlessness. Independent variables MBI: EE, Depersonalization, Personal Accomplishment; and Occupational Status.
- Model 3: Dependent variable (dv): VOCALISE: Confidence. Independent variables MBI: EE, Depersonalization, Personal Accomplishment; and Occupational Status.
- Model 4: Dependent variable (dv): VOCALISE: Demotivation. Independent variables MBI: EE, Depersonalization, Personal Accomplishment; and Occupational Status.

Only significant effects are reported (see Table S1).

Staff expressed greater powerlessness if they had high EE, low personal accomplishment and were direct care staff. Model 2 explained 35% of the variance in the powerlessness variable. Staff were less confident if they had high depersonalization and were direct care staff (these variables accounted for 2% of the variance in confidence. High EE predicted demotivation and accounted for 13% of its variance.

## Discussion

Although in mental health ward service users have highlighted service deficits (specifically, that wards are boring, nontherapeutic and coercive), it is not yet clear why improvements are difficult to embed in these areas. As these are complex settings, a number of influences are likely. Negative appraisals of change by nursing staff in mental health wards may reduce how effectively changes are implemented. Furthermore, as nurses are the largest workforce group in the NHS changes to front‐line services may depend on their cooperation. Steps should be taken to identify potential areas of resistance that can be remediated to increase the chances of success when changes are implemented. In this study, we explored whether burnout (which is a known difficulty for mental health ward staff) and workforce characteristics influenced how staff appraised change in mental health wards.

### Did burnout affect perceptions of barriers to change?

In the wake of increasingly complex care and service limitations, mental health ward staff may be more vulnerable to increasing work pressures and burnout, and may require additional support. In this study, there was a strong, significant effect of burnout on staff perceptions of barriers to change, which explained nearly a quarter (23%) of the variance in the perceptions of barriers to change measure. Clearly addressing burnout in UK front‐line mental health ward staff will be important in facilitating innovation and service developments.

This finding is in line with two other studies conducted in different health settings (one in a neonatal unit in Sweden (Wallin *et al*. 2006) and the other in the US children's mental health services (Aarons & Sawitzky 2006)). The current study provides further evidence that burnout affects how staff perceive barriers to change, in a third service setting (adult mental health wards). These preliminary findings may indicate a more widespread problem, and burnout may be affecting improvements across multiple health services and settings. This must be investigated further and addressed if future changes are to succeed.

Currently, research shows that well‐structured organizations with effective leadership, communication and suitable staffing levels are less likely to have burnt out staff (Hanrahan *et al*. 2010). These broader strategies might usefully feed into more comprehensive, clearly defined and better‐communicated change management plans. As communication appears to be a key factor, it may be beneficial to engage with staff to develop changes that staff find feasible. A staff informed approach has the potential to moderate burnout and negative perceptions towards changes when developments are required.

### Which workforce characteristics affected perceptions of barriers to change?

The literature describes differences in the perceptions of employees (from a variety of workplaces including non‐healthcare settings), depending on their occupational status and age (Bantel & Jackson 1989; Benn *et al*. 2009; Vroom & Pahl 1971). In this study, although age and occupational status were correlated with perceptions of barriers to change, only the effect of occupational status remained significant when both were included in a random‐effects model with burnout. As the effect of occupational status appeared to be independent (explaining more of the variance than burnout alone), exploring why direct care staff are more negative towards changes may assist those wishing to develop implementation strategies. Using the VOCALISE subscales to provide more detail, this study showed that occupational status predicted confidence and powerlessness. Staff that were more senior felt less powerless and more confident than those in more junior positions.

### Why might direct care staff have more negative perceptions of barriers to change?

Direct care staff, who spend much of their time in contact with their client group, had more pessimistic views of change in these data. This finding adds to previous literature, which showed that senior staff were able to comment on the global impact of changes, whereas front‐line staff only commented on local level issues (Benn *et al*. 2009). Staff at the direct care level may be more pessimistic because they are less aware of what motivates centrally driven changes, and may therefore not fully appreciate their scope or relevance.

The perceptions of those in direct care positions may be improved if they are offered a greater level of involvement when changes are introduced. Future research should consider how to engage staff in changes, as innovation and improvements may increase the quality of care offered to service users.

### Limitations and future research suggestions

Given change has been a challenge in mental health wards, this study is an important first step in building a model of variables which impact staff perceptions of barriers to change, using cross‐sectional data. However, this research was conducted in one trust, using data from nursing staff only, and the results will need replication to clarify whether they are generalizable to the wider NHS. Research that is more extensive is also needed to examine whether effects of age, occupational status and burnout affect how staff perceive barriers to change longitudinally, and whether there is a reciprocal relationship between perceptions of barriers to change and burnout.

Random‐effects models are advantageous when studying teams of employees who are likely to have shared perceptions. However, as the random‐effects model computes an average ‘ward effect’, it would be optimal to have high representation from all staff groups. In this sample, some wards had limited representation in senior staff groups. Indeed, ward six includes no band six staff (clinical charge nurses). Inferences from these results to the larger population should therefore be drawn with caution, and clearly, more work is needed in this area.

Although senior staff were more optimistic towards change in this study, a finding that is supported by the literature, staff characteristics accounted for only a small amount of the variance in all the subscales. Furthermore, any significant relationships between the subscales of the MBI and VOCALISE measures showed very small beta coefficients. Future research may therefore consider strategies that aim to improve staff perceptions of barriers to change overall (using the total score of the VOCALISE measure).

## Conclusion

Burnout is clearly a factor that influences staff perceptions of barriers to change. This may partially account for historical difficulties in embedding changes in mental health wards, where morale is lower than other settings. Those wishing to innovate may need to consider how to reduce burnout as part of their implementation strategy. These findings contribute to a currently scant evidence base and have meaningful implications for future implementation projects conducted in mental health wards. Future studies might consider the impact of burnout and occupational status on perceptions of barriers to change in across the NHS more extensively to illuminate whether support is required across the wider system.

## Relevance for Clinical Practice

Although the Nursing & Midwifery Council (2015) Code of Conduct states that nursing staff should practice in line with the best available evidence, there is still a prominent disconnect between front‐line practice and research evidence. Developing methods/practices to support direct care staff in mental health wards, when incorporating planned changes, may improve their perceptions of barriers to change, and reduce resistance when changes are required. It may be productive to consider change strategies that promote front‐line autonomy and develop training that builds confidence. Nursing teams may benefit from identifying which of the nursing roles have the capacity and knowledge to champion innovation, to increase nursing participation in changes. The practice development nurse (PDN) role could be honed to meet this purpose, so that these staff, with direct input from front‐line colleagues, coordinate staff and service development (Table S1).

## Supporting information


**Table S1** ***.*** Random effects models showing how Emotional Exhaustion, Depersonalization, Personal Accomplishment (MBI) and Occupational Status affected VOCALISE (Powerlessness, Confidence and Demotivation)
