# Supplementary material for: Why is change a challenge in acute mental health wards? A cross‐sectional investigation of the relationships between burnout, occupational status and nurses’ perceptions of barriers to change
Source: Int J Ment Health Nurs. 2018 Jul 11;28(1):190–8. doi: 10.1111/inm.12517 (PMC7328713; doi:10.1111/inm.12517)
Supplement: Supplementary file 1 — Table S1 . Random effects models showing how Emotional Exhaustion, Depersonalization, Personal Accomplishment (MBI) and Occupational Status affected VOCALISE (Powerlessness, Confidence and Demotivation) [file INM-28-190-s001.docx]

*Table S1: Random effects models showing how Emotional Exhaustion, Depersonalisation, Personal Accomplishment (MBI) and Occupational Status affected VOCALISE (Powerlessness, Confidence and Demotivation)*

| **Independent variables:** | **Model 1: VOCALISE: Powerlessness (dv)** | **Model 2: VOCALISE: Confidence (dv)** | **Model 3: VOCALISE: Demotivation (dv)** |
| --- | --- | --- | --- |
| **MBI: Emotional Exhaustion** | Coef. β: 0.18  S.E: 0.05  ***p<0.001***  C.I: 0.09 to 0.27 | Not significant | Coef. β: 0.11  S.E: 0.04  ***p=0.005***  C.I: 0.03 to 0.19 |
| **MBI: Depersonalisation** | Not significant | Coef β: 0.23  S.E: 0.09  ***p=0.01***  C.I: 0.05 to 0.40 | Not significant |
| **MBI: Personal Accomplishment** | Coef. β: 0.21  S.E: 0.09  ***p=0.001***  C.I: 0.08 to 0.34 | Not significant | Not significant |
| **Occupational Status** | Coef. β: -2.60  S.E: 1.14  ***p=0.02***  C.I: -4.84 to -0.37 | Coef. β: -3.07  S.E: 1.00  ***p=0.002***  C.I: -5.03 to -1.10 | Not significant |
| *_cons* | Coef. β: 17.54  S.E: 1.25  p<0.001  C.I: 15.07 to 19.99 | Coef. β: 14.75  S.E: 1.31  p<0.001  C.I: 12.19 to 17.31 | Coef. β: 18.57  S.E: 1.13  p<0.001  C.I: 16.34 to 20.80 |
| *N=* | 101 | 100 | 98 |
| *sigma_u* | 0 | 1.96 | 0.49 |
| *sigma_e* | 4.62 | 3.88 | 3.89 |
| *rho* | 0 | 0.20 | 0.02 |
| *Model statistics* | (χ^2^ (4) = 50.81; p>0.001) | (χ^2^ (4) = 31.65; p>0.001) | (χ^2^ (4) = 13.40; p>0.001) |
